# Scaphoid Osteoid Osteoma in a Pediatric Patient: A Case Report and Review of Diagnostic Pitfalls and Management Strategies

**DOI:** 10.1155/cro/4656938

**Published:** 2026-07-29

**Authors:** Charles Spieser, Anthony Achille, Darren Malek, Naem Mufarreh, Gavin Scott, Samuel Shepard, Alfred-John Bayaton, Andrew Malarkey

**Affiliations:** ^1^ Grandview Orthopedic Surgery Residency, Kettering Health Dayton, Dayton, Ohio, USA; ^2^ Ohio University—Heritage College of Osteopathic Medicine, Athens, Ohio, USA; ^3^ Grandview Orthopedic Surgery Residency and Orthopedic Associates of Southwest Ohio, Kettering Health Dayton, Dayton, Ohio, USA

**Keywords:** computed tomography, osteoid osteoma, pediatric wrist, scaphoid, surgical excision

## Abstract

Osteoid osteoma (OO) of the scaphoid is an uncommon entity, particularly in pediatric patients, where diagnosis is often delayed due to its rarity and overlapping presentation with more common wrist pathologies. We report the case of a pediatric athlete with persistent wrist pain initially diagnosed as a scaphoid fracture. Imaging progression from radiographs to MRI suggested fracture, but confirmatory CT revealed a 5‐mm radiolucent lesion with central mineralization, consistent with OO. Surgical excision via volar approach was performed due to intraosseous lesion location and proximity to articular surfaces, with demineralized bone matrix placement and no fixation required. Postoperative recovery was notable for resolution of pain and functional restoration by 2 months. This case highlights the importance of considering OO in atypical locations and the role of CT for definitive diagnosis when MRI is inconclusive. Early recognition can expedite management, reduce morbidity, and prevent long‐term sequelae in pediatric athletes.

## 1. Introduction

Osteoid osteoma (OO) is a benign, bone‐forming tumor defined by a central, highly vascularized nidus surrounded by reactive sclerosis [1]. These lesions measure ≤ 2 cm, differentiating them from osteoblastomas, and account for approximately 10%–12% of benign bone tumors and 3% of all primary bone tumors [2]. They predominantly affect males under the age of 30, with peak incidence during the second decade of life [3].

The classic presentation includes dull, localized pain that worsens at night and is typically relieved by NSAIDs [3]. Radiographically, lesions appear as a small radiolucent nidus with surrounding sclerosis. Although most commonly found in the cortical diaphysis of long bones, atypical locations such as the spine, pelvis, facial bones, and hand complicate diagnosis. Approximately 10% of cases affect the hand, with only 2% occurring in the carpal bones [4]. Scaphoid involvement is particularly rare and often mimics more common pathologies such as occult fracture, tendinitis, or inflammatory arthropathy [5].

Several case reports describe delayed or missed diagnoses in scaphoid OO, with lesions misattributed to De Quervain′s tenosynovitis, scaphoid fracture, or arthritis [6]. This report presents a case of scaphoid OO initially misdiagnosed as a fracture, followed by a review of the diagnostic challenges and treatment strategies relevant to this uncommon presentation. This case report was prepared following the CARE guidelines.

## 2. Case Descriptions

### 2.1. Patient Information

The patient was a 14‐year‐old Caucasian, non‐Hispanic male competitive baseball player who presented with a 2‐month history of intermittent left wrist pain, worse at night and partially relieved by over‐the‐counter NSAIDs. He reported a fall onto an outstretched hand approximately 1 year before the onset of his current symptoms, although any pain associated with that event had resolved before this presentation. He denied any recent trauma, illness, or systemic symptoms. His medical history was unremarkable, with no chronic medical conditions, and his family history was negative for bone tumors or malignancy. Given his high level of athletic activity and repetitive baseball‐related wrist loading during gripping and batting, an occult chronic carpal stress injury, including a chronic scaphoid fracture or nonunion, was considered in the differential diagnosis.

### 2.2. Clinical Findings

On examination, there was focal tenderness over the radial aspect of the left wrist, including the anatomic snuffbox, without swelling, erythema, or deformity. Wrist range of motion was mildly restricted secondary to pain, and grip strength was reduced compared with the contralateral side. Neurovascular examination was normal. No additional provocative maneuvers or special tests were performed.

Initial dedicated four‐view scaphoid radiographs, including posteroanterior, true lateral, oblique, and scaphoid views, were inconclusive. The interpreting radiologist did not identify any osseous abnormality, with good alignment of the carpal rows. A finding with which the senior attending surgeon agreed. Given persistent clinical concern despite negative radiographs, magnetic resonance imaging (MRI) of the left wrist was obtained in accordance with standard diagnostic protocol to rule out an occult nonunion scaphoid fracture.

A follow‐up MRI scan was obtained. Fat‐ and water‐sensitive sequences demonstrated dorsal cortical irregularity at the scaphoid waist with abnormal marrow signal, extensive osseous edema, and surrounding soft‐tissue edema, findings initially interpreted as consistent with a comminuted fracture. However, given the discordance between the MRI findings and the clinical presentation, a dedicated computed tomography (CT) of the wrist was obtained.

**Figure 1:**
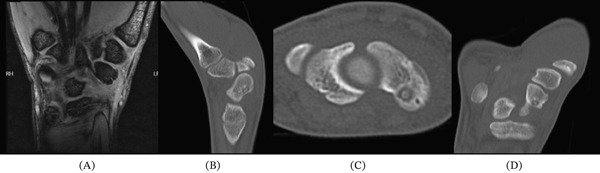
Preoperative MRI and CT scans. (A) Coronal T2‐weighted MRI of the left hand showing increased signal along the radial border of the scaphoid. (B–D) Sagittal, axial, and coronal CT images of the left wrist demonstrating a scaphoid nidus with surrounding sclerosis.

CT revealed a 5‐mm radiolucent lesion with central mineralization in the distal scaphoid, consistent with OO. Subsequent nuclear medicine imaging revealed mild hyperemia in the left wrist, consistent with trauma but without features suggestive of malignancy and nondiagnostic for OO (Figure 1).

Given the lesion′s intraosseous position within the scaphoid and its proximity to articular surfaces, the patient was referred to interventional radiology for consideration of radiofrequency ablation (RFA). However, due to limited percutaneous accessibility and concern for thermal injury, the procedure was deferred. As a result, the patient was scheduled for surgical excision.

### 2.3. Relevant Anatomy

The scaphoid is the largest bone of the proximal carpal row, articulating with the distal radius proximally; the trapezium and trapezoid distally; and the capitate and lunate along its ulnar surface [7]. Approximately 80% of its surface is covered by articular cartilage, leaving limited nonarticular bone available for vascular entry [8]. The scaphoid receives most of its intraosseous blood supply from dorsal branches of the radial artery entering through the dorsal ridge and perfusing the proximal pole in a retrograde fashion [9]. Consequently, disruption of this vascular supply after fracture places the proximal pole at particular risk for ischemia and nonunion [9].

The scaphoid undergoes endochondral ossification from a single ossification center that appears during early childhood and progresses from distal to proximal. Ossification is typically complete at approximately 13 years of age in females and 15 years in males [10]. In younger children, the substantial cartilaginous component of the scaphoid can limit radiographic conspicuity of osseous pathology and contribute to diagnostic difficulty [10, 11]. Although OO does not inherently imply vascular compromise, an intraosseous scaphoid lesion may create diagnostic difficulty and wrist dysfunction in a bone whose structural injury can have clinically consequential consequences.

### 2.4. Surgical Approach and Procedure

A volar approach to the scaphoid was performed under tourniquet control. A small cortical defect was identified along the radial scaphoid margin. Using fluoroscopy and CT guidance, a 0.45‐mm Kirschner wire localized the lesion. A 1 × 0.5 cm excisional debridement was completed, and the defect was filled with demineralized bone matrix. Fixation was unnecessary. The wound was closed in layers and immobilized in a thumb spica splint.

### 2.5. Surgical Follow‐Up

The patient received 2 g of cefazolin within 1 h before incision for perioperative antibiotic prophylaxis. Postoperative QuickDASH (Quick Disabilities of the Arm, Shoulder and Hand) scores were recorded longitudinally.

At 1 week, radiographs showed no interval changes. The patient reported substantial improvement in pain, and the incision was well healed. He remained nonweight‐bearing; the thumb spica splint was removed, and a short arm cast was applied.

At 1 month, the cast was removed, and the patient was transitioned to a wrist brace without activity restrictions. Protruding suture material was noted at the surgical site without drainage. This was considered most consistent with a localized reaction to suture material rather than a true surgical‐site infection. As a precaution, a 10‐day course of trimethoprim–sulfamethoxazole was prescribed. QuickDASH scores continued to improve.

By 2 months, the suture abscess had resolved; the patient reported complete resolution of wrist pain and functional limitation, and radiographs confirmed progressive osseous remodeling consistent with scaphoid healing (Table 1).

**Table 1:** Summary timeline of events.

Discussion	
Date/interval	Event
Month 0	Onset of intermittent left wrist pain during baseball season
Month 2	Initial radiographs—inconclusive
Month 3	MRI—suspected scaphoid fracture
Month 3.5	CT—revealed osteoid osteoma
Month 4	Open surgical excision with demineralized bone matrix placement
1‐week post‐op	Pain improved; transitioned from thumb spica to short arm cast
1‐month post‐op	Suture abscess treated with antibiotics; transitioned to wrist brace
2 months post‐op	Complete pain resolution and return to full activity

This case illustrates the diagnostic challenges posed by OO in the scaphoid. The patient′s age, history of trauma, and MRI findings initially led to misdiagnosis as a scaphoid fracture. Dedicated CT ultimately established the diagnosis by demonstrating a characteristic 5‐mm nidus with central mineralization in the distal scaphoid.

### 2.6. Diagnosis and Diagnostic Pitfalls

OO is classically diagnosed via a triad of localized nocturnal pain relieved by NSAIDs, radiographic evidence of a nidus ≤ 2 cm, and surrounding sclerosis. However, in intra‐articular or small bones such as the scaphoid, these features are often obscured. Standard radiographs are frequently unrevealing due to limited sclerosis or overlying anatomy, with plain films failing to identify the nidus in up to 30% of cases [5]. MRI, although sensitive to edema and soft‐tissue changes, can be misleading. In this case, T2‐weighted MRI suggested a scaphoid fracture, likely reflecting reactive edema. Extensive marrow edema and synovitis may mimic inflammatory or posttraumatic processes and can obscure the nidus entirely [6]. CT remains the gold standard for diagnosis, particularly in atypical or intra‐articular lesions. Nuclear bone scans, although sensitive, are nonspecific and best reserved for difficult cases or when multiple foci are suspected. In this case, the scan showed mild hyperemia, consistent with trauma, but not definitive for OO^12^.

Diagnostic pitfalls include omission of a therapeutic NSAID trial, atypical anatomic locations that mimic more common conditions, anchoring bias following trauma, and overreliance on incongruent MRI findings without confirmatory CT [6].

### 2.7. Treatment Modalities

Conservative management may be appropriate in select cases with mild symptoms, given the potential for spontaneous resolution over several years. However, when pain is persistent, nocturnal, or refractory to NSAIDs, and particularly when functional limitations affect daily activities, surgical or ablative intervention should be pursued. OO may be treated through either open surgical or percutaneous approaches, with selection driven by lesion location, skeletal maturity, and procedural risk profile [12].

RFA is the preferred first‐line treatment for accessible, diaphyseal lesions [13]. It offers high success rates (> 90%), minimal morbidity, and rapid recovery. However, RFA carries risks in juxta‐articular or anatomically complex locations, such as the scaphoid, where thermal injury to cartilage, neurovascular structures, or open physes may occur [14].

RFA is established as the preferred first‐line treatment for OO in accessible, diaphyseal locations, with reported success rates exceeding 90% and a complication rate of approximately 2%–5% across large systematic reviews [15–17]. Critically, subset analyses of intra‐articular and juxta‐articular lesions have demonstrated similarly high success rates (97%) with low complication rates, provided that technique‐dependent precautions are observed [15, 18]. In juxta‐articular locations, risks of thermal injury to articular cartilage, neurovascular structures, and open physes remain a concern, though a series of 59 juxta‐articular cases, including 13 patients with open physes, documented only one instance of articular damage attributable to CT‐guided RFA [18]. Reported cases of successful CT‐guided RFA for scaphoid OO exist [19, 20], suggesting the procedure is technically feasible in this location when institutional expertise is available. In the present case, percutaneous ablation was considered; however, following multidisciplinary consultation with interventional radiology, surgical management was elected based on institutional comfort with the anatomic risk profile. This decision reflects appropriate individualization of care rather than a generalized contraindication to percutaneous ablation in juxta‐articular settings.

Surgical excision remains the treatment of choice for lesions that are intra‐articular, near open physes, or located in small bones where ablation access is limited [14]. It allows for direct visualization, precise nidus resection, and histologic confirmation. Although more invasive, it avoids thermal injury and is associated with lower recurrence rates in select locations. In this case, the combination of skeletal immaturity, articular proximity, and scaphoid location favored open excision [14].

### 2.8. Pediatric Considerations

Open physes and evolving bone morphology in children necessitate caution with thermal‐based interventions [21]. Surgical approaches must preserve perfusion while minimizing disruption to growth potential. Scaphoid vascularity is vulnerable to iatrogenic insult, particularly in its proximal segment [9]. Postoperative immobilization and activity modification must be tailored to facilitate healing without compromising long‐term function.

### 2.9. Limitations and Lessons Learned

This case is limited by short‐term follow‐up and absence of advanced functional outcome scoring beyond the early postoperative period.

### 2.10. Informed Consent

No written informed consent for participation and publication was obtained from the patient or legal guardian. All information in this report, including any images, has been deidentified in accordance with the Health Insurance Portability and Accountability Act (HIPAA) Privacy Rule (45 CFR §164.514) safe harbor method.

### 2.11. Patient Perspective

“Before surgery, my wrist hurt almost every day, especially at night, and I could not play baseball like I wanted. After the surgery, the pain went away, and I was able to get back to playing without any problems.”

## 3. Conclusion

OO of the scaphoid is an uncommon but important consideration in adolescents with persistent wrist pain. This case highlights the value of CT in differentiating OO from more common traumatic or inflammatory conditions when initial imaging is indeterminate. In this skeletally immature patient, open surgical excision was selected based on lesion accessibility and multidisciplinary treatment preference; however, RFA may represent a reasonable alternative in appropriately selected cases. Recognition of atypical presentations and adherence to a structured diagnostic algorithm can expedite appropriate intervention and improve outcomes.

## Funding

No funding was received for this manuscript.

## Conflicts of Interest

The authors declare no conflicts of interest.

## Data Availability

Data sharing is not applicable to this article as no datasets were generated or analyzed during the current study.
